# Supplementary material for: Pillar Growth by Focused Electron Beam-Induced Deposition Using a Bimetallic Precursor as Model System: High-Energy Fragmentation vs. Low-Energy Decomposition
Source: Nanomaterials (Basel). 2023 Nov 6;13(21):2907. doi: 10.3390/nano13212907 (PMC10647607; doi:10.3390/nano13212907)
Supplement: Supplementary file 1 [file nanomaterials-13-02907-s001.zip › nanomaterials-2627993-supplementary.pdf]

Supporting Information

Pillar Growth by Focused Electron Beam Induced Deposition using a Bimetallic Precursor as Model System: High Energy Fragmentation vs Low Energy Decomposition

Robert Winkler,[1] Michele Brugger-Hatzl,[2] Fabrizio Porrati,[3] David Kuhness,[1] Thomas Mairhofer,[4] Lukas M. Seewald,[1] Gerald Kothleitner,[2,4] Michael Huth,[3] Harald Plank,[1,2,4,*] Sven Barth[3,5,*]

[1] Christian Doppler Laboratory—DEFINE, Graz University of Technology, 8010 Graz, Austria.
[2] Graz Centre for Electron Microscopy, 8010 Graz, Austria
[3] Institute of Physics, Goethe University, Max-von-Laue-Str. 1,60438 Frankfurt, Germany
[4] Institute of Electron Microscopy, Graz University of Technology, 8010 Graz, Austria
[5] Institute for Inorganic and Analytical Chemistry, Goethe University Frankfurt, Max-von-Laue-Str. 7, 60438 Frankfurt, Germany
* Correspondence: ; harald.plank@felmi-zfe.at (H.P.); barth@physik.uni-frankfurt.de (S.B.)

Supporting Information 1: additional examples of pillars written with and without beam blur[1] and different currents.

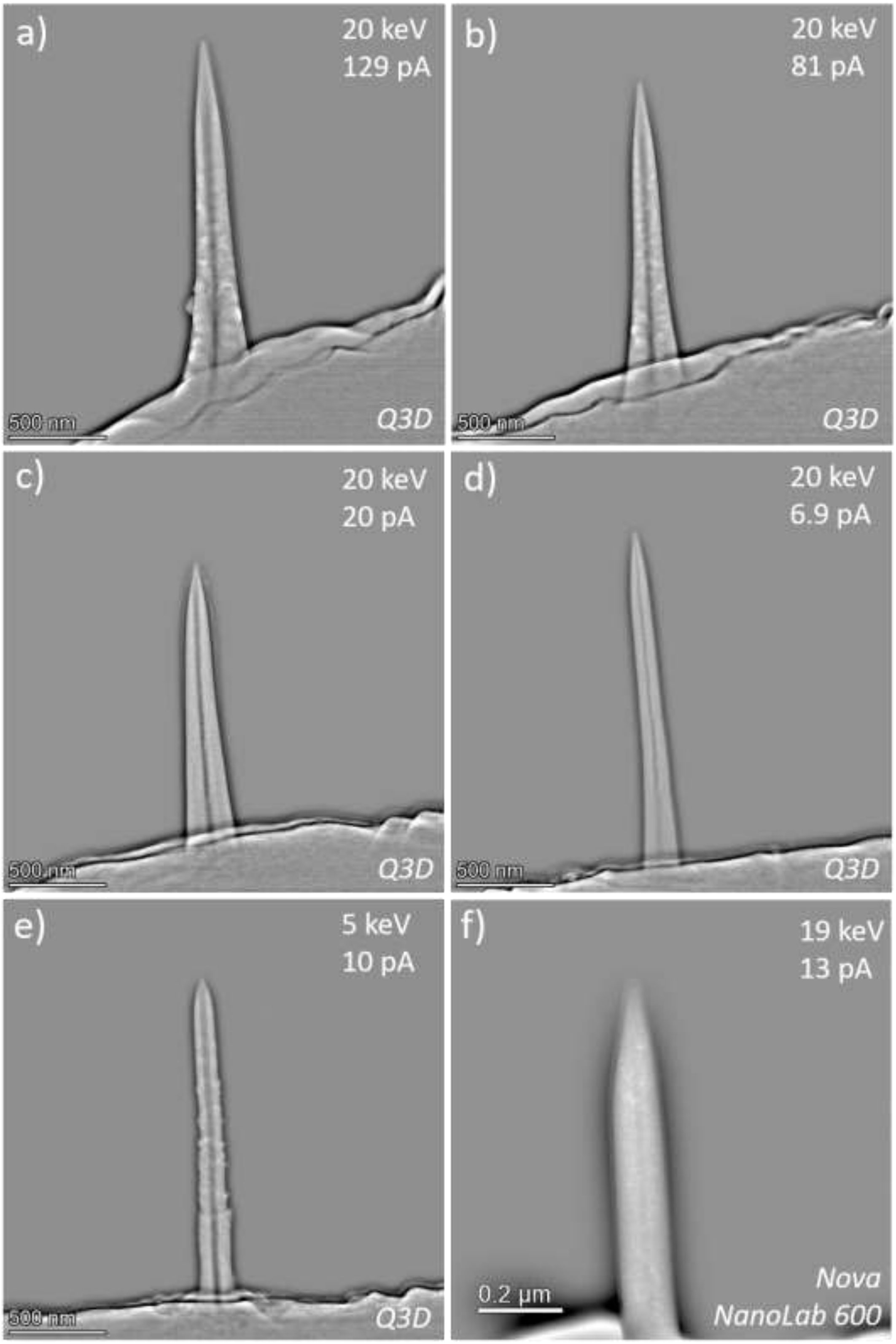

Figure S1: (a-e) Selection of high pass filtered HAADF images of blur-assisted nano-cones and (f) a pillar fabricated under focus conditions. The fabrication parameters are specified top right, the used FIB/SEM microscope is declared bottom right in each image. As evident, all structures reveal the dark core independent on beam, patterning settings and equipment used.

Supporting Information 2: 2D representation of Fe and Co content determined by EDX in the tip region of a nano-cone.

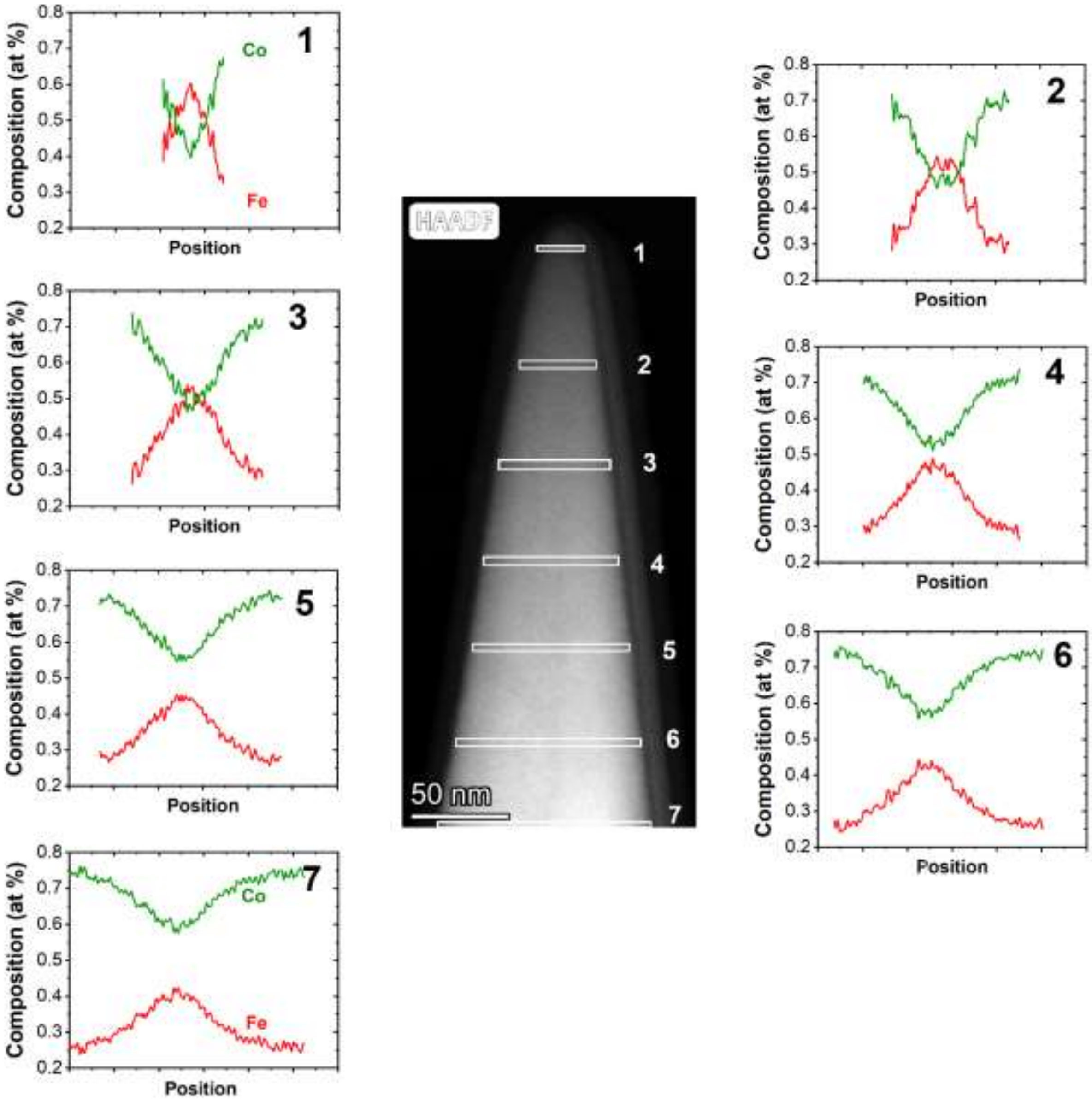

Figure S2: (1-7) show individual line scans of the atomic composition for Co and Fe along the region, as indicated in the central HAADF image. The Fe rich core is observed across the entire structure, which also holds for any other pillar / nano-cone, grown with different parameters as summarized in Figure S1. C and O contents were found (always below 7 at.%) but not shown here for clarity.

Supporting Information 3: Monte-Carlo Electron trajectories simulations for cones.

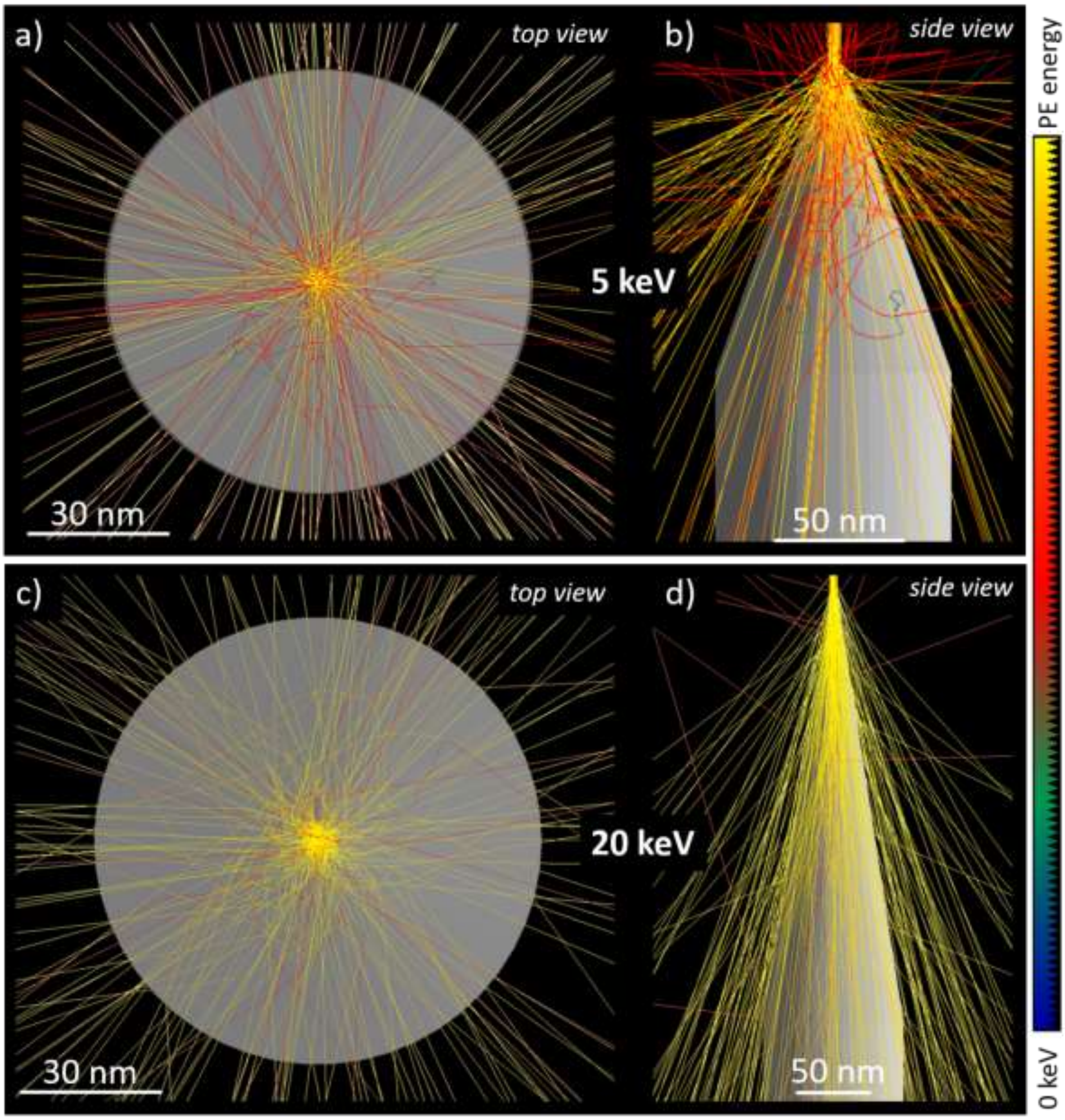

Figure S3: Monte-Carlo electron trajectory simulation (Casino 3.3.0.4)[2] of Co_3FeC cones during FEBID at 5 keV (a,b) and 20 keV (c,d) in top and side views, respectively. Pillar dimension were taken from real experiments (see Figure S1e, f). High energy electrons (yellow trajectories) show a high density at the central axis, while energy losses are reflected by the colour gradient according to the right hand side bar. Backscattered electrons are shown in red and can be identified by the name-giving upward direction.

Supporting Information 4: additional TEM images of ongoing crystallization upon electron-bombardment.

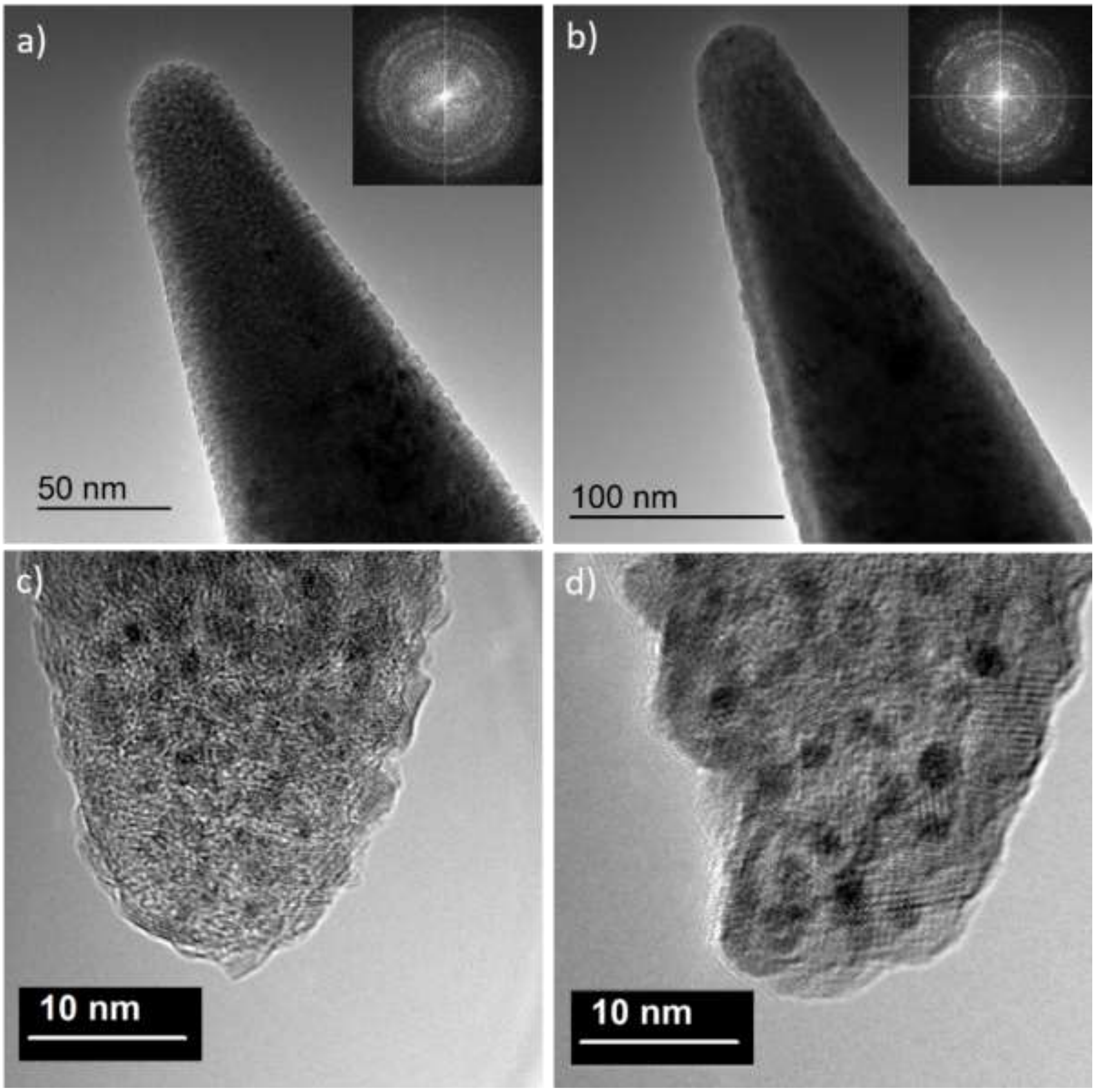

Figure S4: Crystallization effects during TEM long time inspection of an as-deposited nano-cone, similar to electron-beam-curing, as described in the main manuscript. While initial TEM images are shown at the left by an overview (a) accompanied with a FFT and a high-resolution image from the apex (c), the right images (b +d) give the same structure after full TEM analyses, which reveal both, densification (b) and stronger crystallization at the apex (d).

References

[1] R. Winkler, M. Brugger-Hatzl, L.M. Seewald, D. Kuhness, S. Barth, T. Mairhofer, G. Kothleitner, and H. Plank, Nanomaterials **13**, 1217 (2023).

[2] H. Demers, N. Poirier-Demers, A.R. Couture, D. Joly, M. Guilmain, N. de Jonge, and D. Drouin, Scanning **33**, 135 (2011).
